# A Methodology for Network Analysis to Improve the Cyber-Physicals Communications in Next-Generation Networks

**DOI:** 10.3390/s20082247

**Published:** 2020-04-16

**Authors:** David Cortés-Polo, Luis Ignacio Jimenez Gil, José-Luis González-Sánchez, Jesús Calle-Cancho

**Affiliations:** Research, Technological Innovation and Supercomputing Center of Extremadura (CénitS), National Road 521, km 41.8, 10071 Cáceres, Spain; luisignacio.jimenez@cenits.es (L.I.J.G.); joseluis.gonzalez@cenits.es (J.-L.G.-S.); jesus.calle@cenits.es (J.C.-C.)

**Keywords:** 5G, data analysis, mobile networks, network analytic, cyber-physical systems

## Abstract

Cyber-physical systems allow creating new applications and services which will bring people, data, processes, and things together. The network is the backbone that interconnects this new paradigm, especially 5G networks that will expand the coverage, reduce the latency, and enhance the data rate. In this sense, network analytics will increase the knowledge about the network and its interconnected devices, being a key feature especially with the increment in the number of physical things (sensors, actuators, smartphones, tablets, and so on). With this increment, the usage of online networking services and applications will grow, and network operators require to detect and analyze all issues related to the network. In this article, a methodology to analyze real network information provided by a network operator and acquire knowledge of the communications is presented. Various real data sets, provided by Telecom Italia, are analyzed to compare two different zones: one located in the urban area of Milan, Italy, and its surroundings, and the second in the province of Trento, Italy. These data sets describe different areas and shapes that cover a metropolitan area in the first case and a mainly rural area in the second case, which implies that these areas will have different comportments. To compare these comportments and group them in a single cluster set, a new technique is presented in this paper to establish a relationship between them and reduce those that could be similar.

## 1. Introduction

The number of heterogeneous systems connected to the Internet is growing exponentially. In 2022, it is expected that 28.5 billion devices will be connected [1], driven by the appearance of networked devices such as embedded devices, smartphones, smart tables, sensors, radio-frequency identification, and actuators, which use new services and applications. With this emergence, the cyber-physical systems (CPS) acquire a new dimension. CPS represent a new generation of digital systems, which consists of the advanced connectivity, which ensures real-time data acquisition from the physical world and the information feedback from the cyberspace, and the intelligent data management, which adds the analytical and computational capability that constructs the cyberspace [2]. The increment of a huge number of CPS devices onto cellular networks will quickly overload the network, causing performance degradation to cellular users on one hand, and on the other hand, preventing CPS to take advantage of global connectivity, ubiquitous coverage, reliability, and security features of cellular networks [3].

To provide the advanced connectivity to CPS, 5G technology is considered as one of the most important, because it integrates entities, communications, and control technologies [4,5], as 5G supports enhanced mobile broadband communications, ultra-reliable and low-latency communications (URLLC), and massive machine-type communications (mMTC) [6,7]. In this environment, URLLC and mMTC in 5G are closely related to CPS. In addition, network operators must improve the maintenance of the network and assure the availability of resources, using network traffic monitoring systems to check, analyze, and manage the network. The information collected describes call volume, calling, and location patterns of the devices, which can be analyzed to extract knowledge about it [8]. This information is also useful to analyze the behavior and consumption patterns of the interconnected devices. When a device is connected to a network, it must periodically reports its presence to the network or connection to the Internet. This report is useful to the carrier to obtain detailed information about the spatial-temporal localization included in a Call Detailed Record (CDR) [9]. These data sources are increasing its importance because they contain valuable information about the network usage, even more so when the number of connected devices is growing exponentially [10]. The information contained in a CDR is different from other data sets, which contain fine-grained data, and it presents a variety of challenges to be resolved [11]. Besides, the location of the devices is only recorded when they use the network, causing, altogether the sparseness of the spatial CDR data, because of the device mobility cannot be tracked. Furthermore, in some cases, data is aggregated in patterns, like a grid [12], complicating the analysis.

In general, geolocated data sets fit the network information to a concrete space and time, being considered, each instant, a three-dimensional data cube where the first two dimensions are the *X* and *Y* spatial components and *Z* the characterization of the area based on the network information collected. Then, the problem could be divided depending on how we consider the data to be organized. Two main models can be considered: a linear model [13] and a nonlinear model [14]. This work is focused on the linear model approach, considering each sub-area characterization a linear combination of different unknown network comportments, representing unique descriptions of a particular activity in the network.

The main contributions of this paper are summarized below.

- A methodology is presented to analyze real network data sets, extract the comportment of each analyzed area and acquire the knowledge of the network users’ behavior.
- Two data sets are analyzed in order to prove the methodology introduced in [15]. These real data sets are provided by Telecom Italia and cover different areas of Italy. One is located in the Trento province in Italy, which is a mainly rural area and covers a larger zone than the other analyzed data set. The second one is located in the city of Milan, which covers a metropolitan zone; the area is smaller and the number of connected devices is bigger.
- A new technique is developed to compare the comportments obtained in both analyses, grouping them in a single cluster set and reducing those that are similar, making more accurate the methodology presented in this work.

The rest of this paper is organized as follows. Section 2 summarizes the methodology used. A set of experiments based on the described methodology are introduced in Section 3 in order to provide an analytical comparison. Finally, Section 4 contains some conclusions and future research lines.

## 2. Proposed Methodology

**Figure 1:**
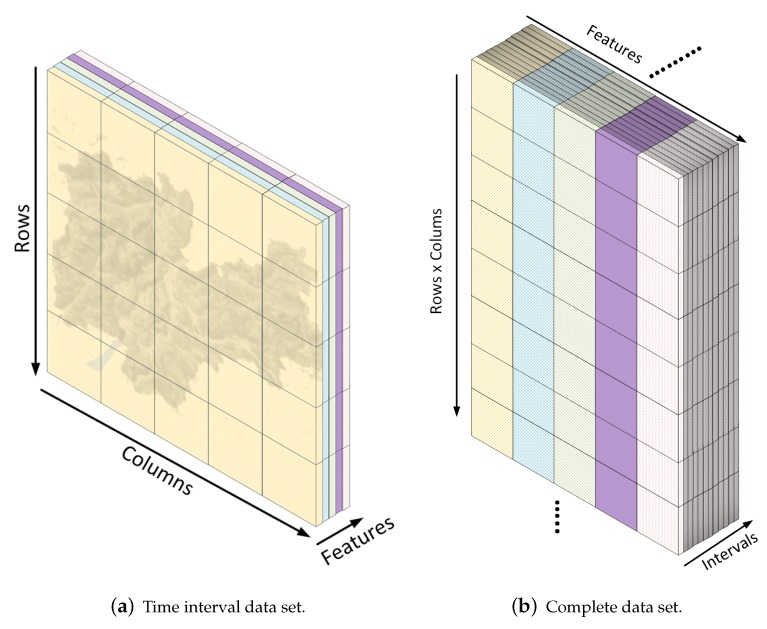
Panel (**a**) depicts a graphical representation of a three-dimensional data cube of the Trento province data set, where one dimension covers the set of network characteristics in each cell located by the other dimensions which represent the geographic information. Panel (**b**) represents the complete data set as a three-dimensional data cube, where it is represented each reshaped time interval.

Nowadays, mobile communications provide a large amount of data about the state of the network and the communication [16], as different services are used under the same infrastructure simultaneously, forcing the companies to track and analyze each service provided to the devices to analyze the usage of the network. Considering this information as a whole, instead of a single service, where a geolocation reference is added to the information, different conclusions might be inferred, treating the information provided by each service as a component of a vector which describes the comportment of the devices connected to the wireless network in a particular area. As a consequence, it is possible to represent this information as a three-dimensional data cube (Figure 1a), where the first two dimensions represent the spatial coordinates in a two-dimensional space and the last one captures the comportment of the network in that particular location. In addition, taking into account the time variable of the data set, the original data cube must be reshaped representing a single interval. The union of every single interval builds a new data cube describing the evolution of every cell in time as is shown in Figure 1b.

Considering that the physical infrastructure has a limited number of comportments, it is assumed that the comportment of each sub-area, defined by the dimensions of an imaginary grid overlapped to the geographical representation of the network, is a combination of this limited information. Two models can be applied to characterize sub-areas in the data cube. The nonlinear approximation considers that the relationship between elements is not linear. The linear approximation, which is applied in this work, considers that the value of each cell is a uniquely weighted linear combination of a subset of these comportments. The linear model can be expressed in mathematical form as shown in Equation (1),

(1) Y=Uα+e

where *Y* is the complete data set, *U* are the comportment arrays in matrix form, α are the weights matrix, and e∈R is a matrix that represents the error introduced during the process. The methodology uses the Orthogonal Projections algorithm (OSP) [15] to select the reference vectors of the comportments. In order to establish a relationship between each class defined by a comportment and every sub-area, $y_{n}$, in the data set *Y*, a Euclidean distance-based technique is performed, assigning each sub-area to the closest comportment $c_{l}\in U$.

Algorithm 1 describes the iterative algorithm to select those extreme points in the *f*-dimensional space formed by the feature arrays collected for each sub-area of the data cube.
**Algorithm 1** Pseudocode of **Orthogonal Subspace Projection** algorithm.**INPUTS**: $Y_{n\times f}\in R^{t}$**$x_{0}$ = HMP**                                                            ▹ First Step$U=[x_{0}\;|\;0\;|\;\cdots \;|\;0]$**for**i=1toi<t−1**do**  $p\;=\;P_{U}^{⊥}Y$                                                  ▹ Second Step  $j\;=\;argmax_{1,\cdots ,r}\to max\;=\;p\left[j,:\right]$▹ Third Step  U[i+1,:]=Y[j,:]
**end for**
where *Y* is the input of the algorithm. First, the algorithm looks at the highest module point (HPM) in the analyzed data cube. This will be the first target of the set *U*. Iteratively, $P_{U}^{⊥}$ is calculated based on the columns of the matrix *U*. This result in conjunction with *Y*, which will be the vector *p* orthogonal to the subspace *U*. In the third step, the index with the maximum argument will point to the next target of the algorithm. The process ends when all targets are selected.

**Figure 2:**
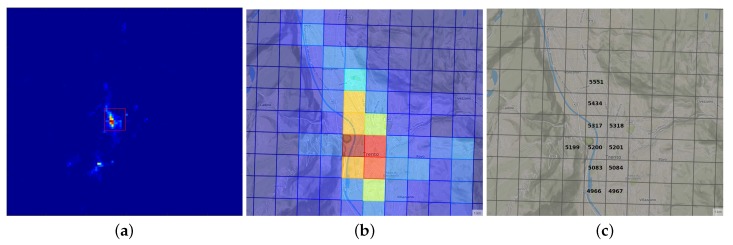
Panel (**a**) shows the classification map resulted after the Euclidean distance classification process over one interval of the data set. Panel (**b**) shows the urban area of Trento which has been zoomed in order to appreciate the difference between urban areas and rural areas within this interval. Panel (**c**) shows the cell identification in the city center of Trento. (**a**) Classification map of the Trento province; (**b**) Projection of the city center of Trento classification; (**c**) Cell identification of the city center of Trento.

To represent the results obtained, the *U* matrix is sorted by module, ordering the comportments from lowest to highest usage of the network. An example of a resulting classification in a cluster map is illustrated in Figure 2a,b.

## 3. Experimental Results

### 3.1. Description of the Used Data Sets

The data set was published by Telecom Italia and SpazioDati in 2014 through the first Big Data Challenge. The purpose of this challenge was to promote technical approaches to telecommunications data sets. After the challenge, the data set was released [12], including telecommunications and social data of Milan and Trento province, describing urban and rural areas. The data set covers 2 months (from November to December 2013). The information provided by the data set includes the sent–received SMS, in which a new registry is generated each time a user sends or receives an SMS. Additionally, the data set includes information about the calls, in which a new record is generated each time a user does or receives a call, and finally the Internet, where a record is generated each time a user starts or ends an Internet connection. This record only will be generated if the connection last form more than 15 min or more than 5 MB is transferred.

As mentioned before, this work is focused on the Trento province data set. The province is divided using a grid of square cells of 1000 m per side, covering an area of 6000 km${}^{2}$ with a total population of ~0.5 million. In comparison with Milan data set [15], it covers more extensions, the cells are larger, and it also introduces an irregular pattern in the disposition of the information collected by adjusting to the shape of the province. The data is obtained in 10 min intervals to collect the aforementioned information from each cell in the grid as an activity level.

To analyze the entire data set, including all intervals, the information is organized in several data cubes of the same area during a period of time, as is shown in Figure 1b, allowing us to perform a set of experiments through the period of time recorded.

### 3.2. Analysis Conducted of the Data Set

The analysis process started using the same methodology as presented in [15], choosing the same date (Thursday, 14 November 2013) for Milan and the Trento province data sets. On this date, various intervals (8:00 h, 15:00 h and 20:00 h) have been analyzed to cover different network behaviors during the day. Each day in the data set is divided into 144 intervals of 10 min obtaining five characterizing vectors from each. A set of classes is extracted from each interval, and in order to make a fair daily classification, the vectors obtained are combined to create a single set of 720 classes, which represent all the comportments in the selected date, following Equation (2),

(2) $$CS=\underset{1\leq i\leq N}{⋃}U_{i}$$

where CS is the final cluster set, $U_{i}$ is the cluster set extracted in each interval, and *N* is the total number of intervals in which the data set is divided.

**Figure 3:**
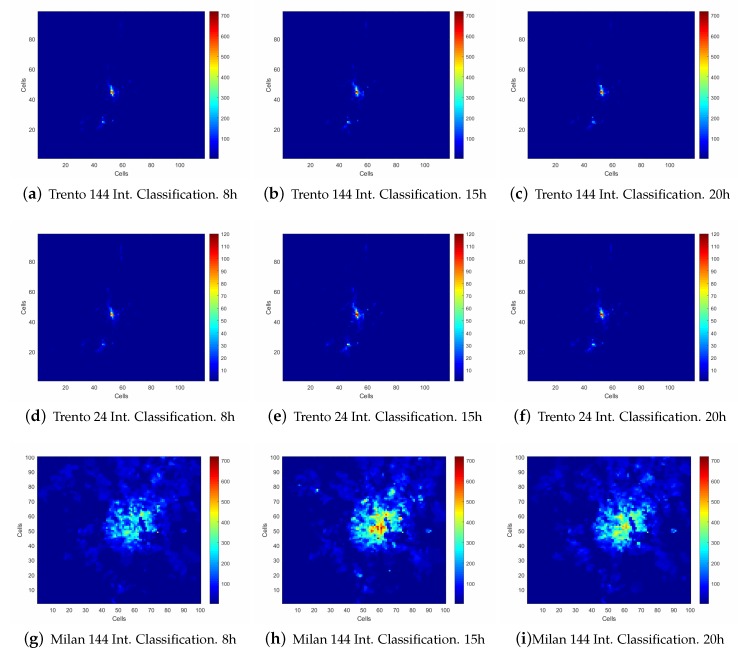

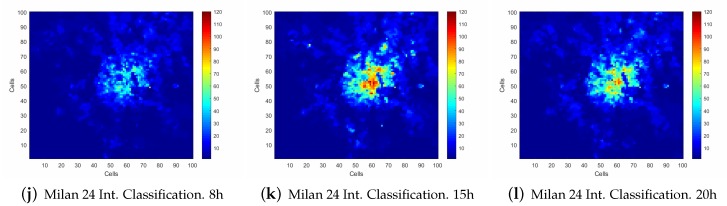
Heat map representation of the network usage in three different hours of the day: 8:00 h, 15:00 h, and 20:00 h. (**a**–**c**) The classification performed in the Trento province using the data set of one day divided into 144 intervals of 10 min. (**d**–**f**) The classification performed into the Trento province data set using one day divided into 24 intervals of 1 h. (**g**–**i**) The classification performed in one day divided into 144 intervals of 10 min using the Milan data set. (**j**–**l**) The classification performed to the Milan data set on the same day grouped into 24 intervals of 1 h.

In Figure 3a–c, the classification results for the intervals are shown, using the comportments extracted in the analyzed date. As could be observed, almost 99.5% of the Trento province is classified with lower network usage profile classes, considering these classes in comparison with the rest of the cluster set. As this scenario is a mountain area, it is perfectly reasonable to assume these classes represent the main rural area. Figure 3g–i shows the classification results from the Milan data set using the same intervals as analyzed in the Trento data set. In this case, almost 98% of the area in the city of Milan is classified with the lower network usage profile classes, being these cells located in the rural area. Both data sets locate the main activity of the network at the downtown of both cities as is shown in Figure 3e,k. As could be observed at 8:00 h, in Figure 3a,g the highlighted zones match with the metropolitan area of Milan and Trento and some surrounding towns, which has higher network usage than the rural areas analyzed. Taking into account the complete usage map, the network is classified mostly in a lower profile compared with the network usage at 15:00 h displayed in Figure 3b,h. In these figures, the city center is highlighted in relation to the rest of the metropolitan area, classified with one of the highest network usage profiles within the whole day. In the 20:00 h analysis described in Figure 3c,i, similar comportments are shown where the highest network usage profile is located in the city center, but classified as a mid-network usage profile.

The description made for the network usage is fine-grained for every cell, but the number of classes that classifies the data set in a single day could be considered too high. In order to provide a better solution to this issue, a reduction in the time component is performed grouping the intervals hourly. This process reduces the number of classes from 720 to 120, shorting the complexity of the problem as is shown in Figure 3d–f, which depicts the Trento province classification, and Figure 3j–l, which describes the comportments classified in the area of Milan. A comparison between images, assures that by using the 144 interval classification the information displayed is clearer and variations between similar network usage profiles can be depicted by the oscillation in the color of the class.

**Figure 4:**
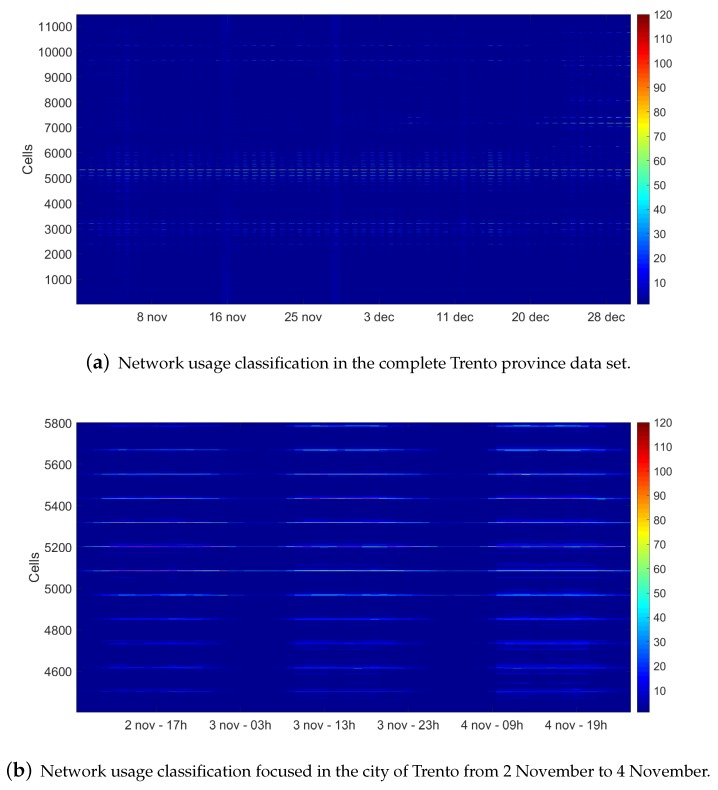

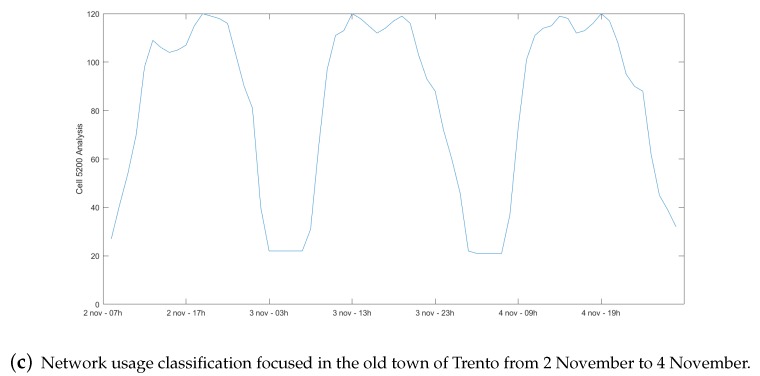
(**a**) Graphical representation of the network usage by hour in the Trento province data set for complete two months (November to December of 2013). (**b**,**c**) Detailed classification of the city of Trento and its old town during a particular period from 2 November to 4 November.

Using the new cluster set, it is easier to describe the whole Trento province data set and analyze the comportments as shown in Figure 4a. In the figure, the evolution of the comportment of the cells in time is described. As could be observed, the lower level network profile corresponds with the rural area which does not modify the comportment over time. At around cells 5000–5300, the cells are classified as the higher network usage comportment of the data set. These cells correspond with Trento downtown (Figure 2c) and describe the comportment of the city during the two months analyzed. Figure 4b zooms to the aforementioned cells analyzing various days. The comportment follows a day–night pattern [17] distinguishing the differences between day and night periods. Particularly, the analysis of a single cell, located in the old town of Trento, shows that this cell registers one of the highest network usage profile of the data set. The evolution of the comportments in this cell are shown in Figure 4c. As expected, a day–night pattern is visible from 7:00 h to 20:00 h, being around 15:00 h the highest network usage. Comparing this metropolitan area with a rural one, even the lowest usage profile is classified in the metropolitan area, and it can be observed that the usage of the network is higher than in the rural area. It is also possible to observe that on weekends the network usage trend remains but stays at higher levels until 22:00 h [10].

**Figure 5:**
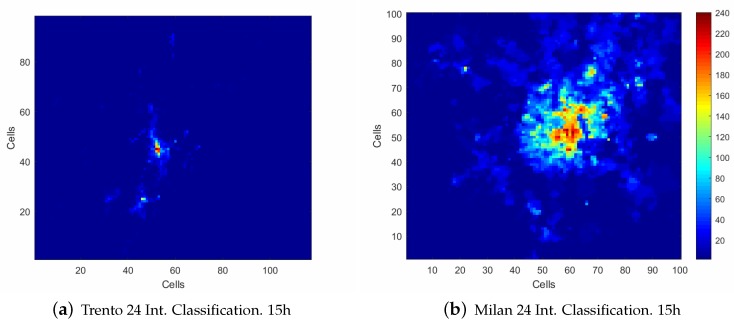
Heat map representation of the network usage at 15:00 h. (**a**) The classification made to the Trento province data set using the unified vectors. (**b**) The classification performed to the Milan data set using the same unified classes. Both data sets are grouped hourly.

The analysis presented in Figure 3 compares the classes obtained using the OSP algorithm from the Milan and Trento province; however, these classes are not interrelated, with only the scale of intensity of the network usage known within each of the sets. To establish a relationship between the levels of network usage in the data sets, merging of both comportment sets into one is performed. The new comportment set contains 240 classes. Figure 5 shows the classification with the new comportment set. As could be observed, both figures are similar to the classification made with 120 classes shown in Figure 3e,k, respectively, maintaining the same relation between the cells in the same data set. With the proposed union, both classifications, shown in Figure 5a,b, are now related by the intensity of the network usage, because every comportment is sorted by module, ordering the set from lower usage profile to a higher usage profile of the network. The new classification of the Milan and Trento province data sets by this new comportment set implies that some of them could be similar, increasing the duplicity of the classes, and by extension, the error. To avoid this, a new technique is applied to group similar comportments using the angle between vectors (ABV) Equation (3), which measures the similarity between two vectors,

(3) $$\theta \left(u,v\right)=\arccos\left(\frac{u\;v}{\left\|u\|\|v\right\|}\right)$$

where θ is the angle between the analyzed vectors *u* and *v*. ‖*u*‖ is defined as the norm of the vector *u* and can be calculated using the formula $\|u\|=\sqrt{u_{1}^{2}+u_{2}^{2}+\cdots +u_{n}^{2}}$, where *u* is an *n*-dimensional vector.

The θ value must be between 0≤θ≤π. To analyze the similarity, a threshold must be defined to remove the similar vectors. In this work, the maximum threshold is set up as 2.5 degrees, and those vectors that are similar using the ABV approach are unified to reduce the number of possible comportments in the classification. In this case, after joining both sets of classes, the lowest module comportment is chosen to be a reference of the *n*-dimensional coordinates system and the angle to the rest of comportments is measured. Sorting the set by this value, the comparison between the reference and the other vectors can provide which ones can be removed from the set based on a degree threshold value $\theta_{0}$, set to this purpose. To measure the error produced by the reduction of the cluster set in the classification result, the Root Mean Square Error (RMSE) measurement is used as is described in Equation (4),

(4) $$RMSE\left(Y,Y^{\prime }\right)=\sqrt{\frac{\sum_{i}^{F}{\left(y_{i}-y_{i}^{\prime }\right)}^{2}}{F}}$$

where *Y* is the original data set and $y_{i}$ is one of the vectors in *Y*. $Y^{\prime }$ is the reduced data set, and $y^{\prime }$ is one of the vectors contained in $Y^{\prime }$.

**Figure 6:**
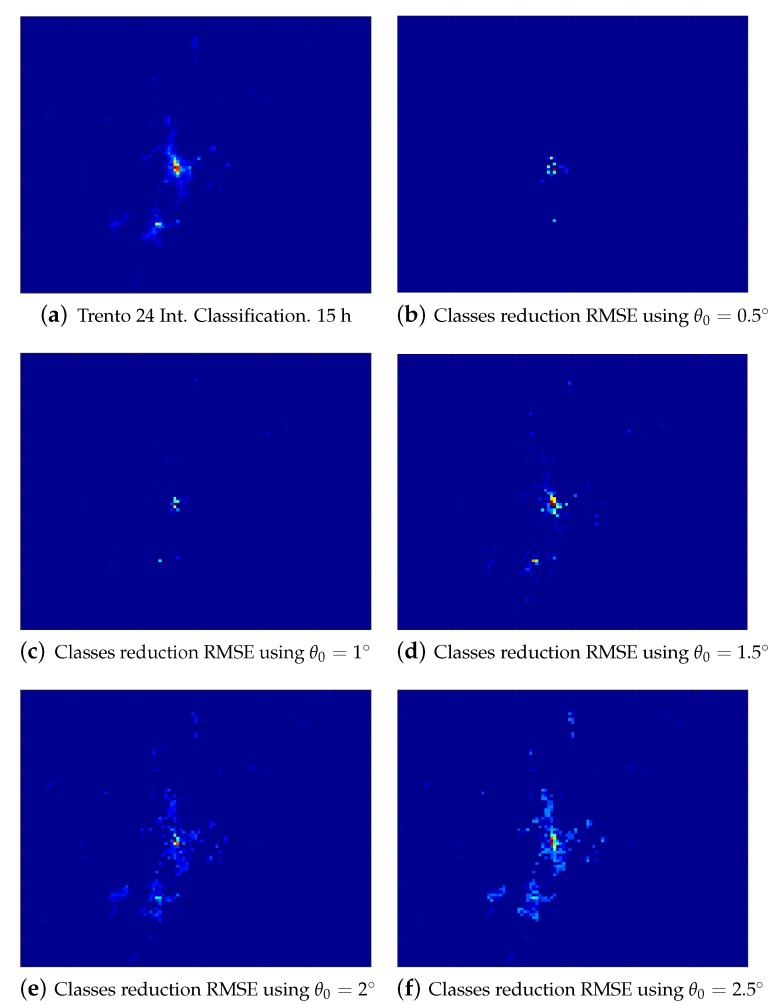
(**a**) The original classification map. (**b**–**f**) The approximated Root Mean Square Error (RMSE) values after comparing the classifications obtained using several threshold (θ) values for the similarity reduction performed in the cluster sets union.

Figure 6a depicts the original classification of the Trento province at 15:00 h in order to have a reference for the error maps displayed from Figure 6b–f. These figures show the approximate error in the cells, classifying the data using the original set of comportments or by means of the reduced one. It can be seen how the urban areas have higher error values as we increase the threshold value $\theta_{0}$, as there exist cells where the network behavior varies more. Table 1 shows the mean RMSE value obtained along with the number of classes reduced in the cluster set. As could be observed with $\theta_{0}=0.5{}^{\circ}$ in Figure 6b, the number of classes is reduced from 240 to 179, this means that around 25% of similar comportments are unified. When the value of $\theta_{0}$ is set to 1.5° it already begins to affect areas outside the center of Trento. Conversely, Figure 6f sets the number of classes to 27, an almost 90% of reduction, corresponding an important loss information in the key areas. The RMSE value obtained using different thresholds up to 2.5° implies that most of the comportments are located in the Trento city area and also the proximity between comportments is in the range of tenths of a degree. Finally, the number of extracted comportments from the Milan data set is around 50% in every reduced comportment set, as shown in Table 1. This means that there are some comportments found in the Milan data set, which describes the metropolitan and suburban areas, that are similar in the Trento province data set which defines a bigger zone, mainly rural.

## 4. Conclusions

Future 5G networks will benefit cyber-physical systems to facilitate the interconnection of a massive number of devices to the network seamlessly. To take advantage of the connectivity, network operators and service providers must adapt to this new paradigm analyzing the network and its behavior. Thanks to the information extracted from next-generation networks, like 5G, the information can be analyzed and used to deploy virtual functions in the network, manage physical and virtual Radio Access Networks and model the spectrum usage, as well as develop flexible services in the network. For this reason, this work is focused on a research field that, in a short time, will be a key field in the deployment of intelligent networks and application services. This article presents a novel methodology that analyzes and classifies real network traffic by using a real CDR data set from Telecom Italia. The information obtained from the real data set corresponds with two differentiated locations: the Milan data set, which describes a big city, with smaller cells and a higher number of user per cell, and the Trento province data set, which describes a vast rural extension, with a medium city and a lower number of users per cell. The proposed methodology, which analyzes geolocated mobile information contained in data cubes, has demonstrated that there are characteristics that can be inferred from the comportment of the network. This work has studied the patterns of the data set, analyzing the usage profile of the network classified in a particular scenario, i.e., a big city like Milan, can be found in other scenarios, like those described in Trento province. This is because there are characteristics that can be inferred from the comportment of the network due to the spatio-temporal relationship with the mobile network usage. This relationship is useful to network managers in controlling the connectivity of a huge number of devices, as is required in cyber-physical systems.

## Figures and Tables

**Table 1:** Mean RMSE values calculated after comparing the original classification with the reduced set of comportments obtained using different values for ($\theta_{0}$).

	$\theta_{0}=0.5^{\circ }$	$\theta_{0}=1^{\circ }$	$\theta_{0}=1.5^{\circ }$	$\theta_{0}=2^{\circ }$	$\theta_{0}=2.5^{\circ }$
RMSE	0.016	0.052	0.116	0.295	0.439
Number of Classes Decreased	61 (25.42%)	127 (52.92%)	176 (73.33%)	190 (79.17%)	213 (88.75%)
Number of Classes Extracted
from the Milan Data Set	93 (51.95%)	59 (52.21%)	31 (48.44%)	28 (56%)	12 (59.26%)

## References

[1] Cisco Systems, Inc. (2019). Cisco Visual Networking Index: Global Mobile Data Traffic Forecast Update, 2017–2022. www.cisco.com.

[2] Lee J., Bagheri B., Kao H. (2015). A Cyber-Physical Systems architecture for Industry 4.0-based manufacturing systems. Manuf. Lett..

[3] Atat R., Liu L., Wu J., Ashdown J., Yi Y. (2019). Green Massive Traffic Offloading for Cyber-Physical Systems over Heterogeneous Cellular Networks. Mob. Netw. Appl..

[4] Atat R., Liu L., Chen H., Wu J., Li H., Yi Y. (2017). Enabling cyber-physical communication in 5G cellular networks: Challenges, spatial spectrum sensing, and cyber-security. IET Cyber-Phys. Syst. Theory Appl..

[5] Varga P., Peto J., Franko A., Balla D., Haja D., Janky F., Soos G., Ficzere D., Maliosz M., Toka L. (2020). 5G support for Industrial IoT Applications—Challenges, Solutions, and Research gaps. Sensors.

[6] Gupta A., Jha R.K. (2015). A Survey of 5G Network: Architecture and Emerging Technologies. IEEE Access.

[7] Siddiqi M.A., Yu H., Joung J. (2019). 5G Ultra-Reliable Low-Latency Communication Implementation Challenges and Operational Issues with IoT Devices. Electronics.

[8] Qiao Y., Xing Z., Fadlullah Z.M., Yang J., Kato N. (2018). Characterizing Flow, Application, and User Behavior in Mobile Networks: A Framework for Mobile Big Data. IEEE Wirel. Commun..

[9] Von Mörner M. (2017). Application of Call Detail Records—Chances and Obstacles. Transp. Res. Proced..

[10] Naboulsi D., Fiore M., Ribot S., Stanica R. (2016). Large-Scale Mobile Traffic Analysis: A Survey. IEEE Commun. Surv. Tutor..

[11] Cheng X., Chen C., Zhang W., Yang Y. (2017). 5G-Enabled Cooperative Intelligent Vehicular (5GenCIV) Framework: When Benz Meets Marconi. IEEE Intell. Syst..

[12] Barlacchi G., Nadai M.D., Larcher R., Casella A., Chitic C., Torrisi G., Antonelli F., Vespignani A., Pentland A., Lepri B. (2015). A multi-source dataset of urban life in the city of Milan and the Province of Trentino. Sci. Data Nat..

[13] Roman S., Axler S., Gehring F.W. (2005). Advanced Linear Algebra.

[14] Khalil H.K. (2002). Nonlinear Systems.

[15] Cortés-Polo D., Jimenez L.I., Calle-Cancho J., González-Sánchez J.L. (2019). A novel methodology based on orthogonal projections for a mobile network data set analysis. IEEE Access.

[16] Andrews J.G., Buzzi S., Choi W., Hanly S.V., Lozano A., Soong A.C., Zhang J.C. (2014). What will 5G be?. IEEE J. Select. Areas Commun..

[17] Thompson K., Miller G.J., Wilder R. (1997). Wide-area Internet traffic patterns and characteristics. IEEE Netw..

